# The complete mitochondrial genome of Wonwhang (*Pyrus pyrifolia*)

**DOI:** 10.1080/23802359.2017.1413300

**Published:** 2017-12-11

**Authors:** Ho Yong Chung, So Yun Won, Sang-Ho Kang, Seong-Han Sohn, Jung Sun Kim

**Affiliations:** Department of Agricultural Biotechnology, National Institute of Agricultural Sciences, Rural Development Administration, Jeonju, Republic of Korea

**Keywords:** *Pyrus pyrifolia*, mitochondrial genome, phylogenetic analysis, PacBio RSII P6-C4 sequencing

## Abstract

This is a *de novo* assembly and annotation of a complete mitochondrial genome from *Pyrus pyrifolia* in the family Rosaceae. The complete mitochondrial genome of *P. pyrifolia* was assembled from PacBio RSII P6-C4 sequencing reads. The circular genome was 458,873 bp in length, containing 39 protein-coding genes, 23 tRNA genes and three rRNA genes. The nucleotide composition was A (27.5%), T (27.3%), G (22.6%) and C (22.6%) with GC content of 45.2%. Most of protein-coding genes use the canonical start codon ATG, whereas *nad1*, *cox1*, *matR* and *rps4* use ACG, *mttB* uses ATT, *rpl16* and *rps19* uses GTG. The stop codon is also common in all mitochondrial genes. The phylogenetic analysis showed that *P. pyrifolia* was clustered with the *Malus* of Rosaceae family. Maximum-likelihood analysis suggests a clear relationship of Rosids and Asterids, which support the traditional classification.

Pears (*Pyrus* spp.), which belong to the family of Rosaceae, have been cultivated in East Asia, and in other countries such as India, Australia, New Zealand, and the United States for more than 3000 years, and are one of most important fruit crops in temperate regions (Bell 1991). *Pyrus* accessions can be classified into three groups. One group comprises *P. brestchenidri*, *P. pyrifolia,* and *P. ussureinsis* in the commercial cultivation of Asia. And also, there is European pear (*P. communis*), the other rootstock group contains *P. betulaefolia* and *P. calleryana*. Asian pears are classified in first of these groups and have a sweet flavour, rich juice, and crisp flesh (Kim 2016).

The plant sample of Wonwhang (Kim et al. 1995) was collected from Naju (Latitude: 35° 01′25.6″N, Longitude: 126° 44′38.4″E), Republic of Korea and representatively identified by Dr Y. K. Kim of the Pear Research Station, National Institute of Horticultural & Herbal Science, Rural Development Administration. Total genomic DNA was extracted from 1 g of young leaves using 1 ml aqua phenol (Mpbio 11AQUAPH) following Kim et al. (2006) with minor modifications. The complete chloroplast genome of Wonwhang, *Pyrus pyrifoia*, was assembled and analyzed by *de novo* assembly using whole-genome sequencing data (Chung et al. 2017). Sequencing was performed using a PacBio RSII platform in P6-C4 chemistry (Pacific Biosciences, Menlo Park, CA) at DNA Link (http://www.dnalink.com/korean/) in Seoul, Republic of Korea. The Single Molecule, Real-Time (SMRT) sequencing, PacBio RSII P6-C4 sequencing, was conducted using the smrtmake assembly pipeline (Chin et al. 2013). We performed the *de novo* assembly using Canu (Koren et al. 2016) to produce a complete mitochondrial genome. And ribosomal RNAs (rRNAs) and transfer RNAs (tRNAs) were predicted using RNAmmer 1.2 (Lagesen et al. 2007) and tRNAscan-SE 1.4 (Lowe and Eddy 1997). The annotated mitochondrial genome sequence using MITOS (Bernt et al. 2013) was deposited into GenBank under the accession number KY563267.

The circular genome of *P. pyrifolia* is 458,873 bp in length, which consists of 39 protein-coding genes (PCGs), 23 tRNAs and three rRNAs. The nucleotide contents of A, T, G and C in mitochondrial genome were 27.5%, 27.3%, 22.6% and 22.6%, respectively. Most of PCGs contained ATG as the start codon, while *nad1*, *cox1*, *matR* and *rps4* began with ACG, *rpl16* and *rpl19* with GTG and *mttB* started with ATT. All PCGs were terminated with the typical stop codons. The 23 tRNA genes ranged from 71 bp and 88 bp in length. The *rrn5*, *rrnS* and *rrnL* were 119 bp, 1782 bp and 3131 bp, respectively.

Phylogenetic analysis was performed using the complete mitochondrial genomes of twelve members including seven Rosids, one Asterids and three Amaranthaceae family, *Oryza sativa* in Monocut was used as outgroup. Sequences for each mitochondrial genome were aligned using ClustalW, and a maximum-likelihood phylogenetic tree was reconstructed using MEGA7 (Kumar et al. 2016) with 1000 bootstrap replicates (Figure 1). The phylogenetic tree showed that mitochondrial genome of *P. pyrifolia* was clustered with *Malus* genus and belonged to Rosids clade. The complete mitochondrial genome of *P. pyrifolia* will provide essential and important DNA molecular data for phylogenetic and evolutionary analysis for Asian pears.

**Figure 1.**
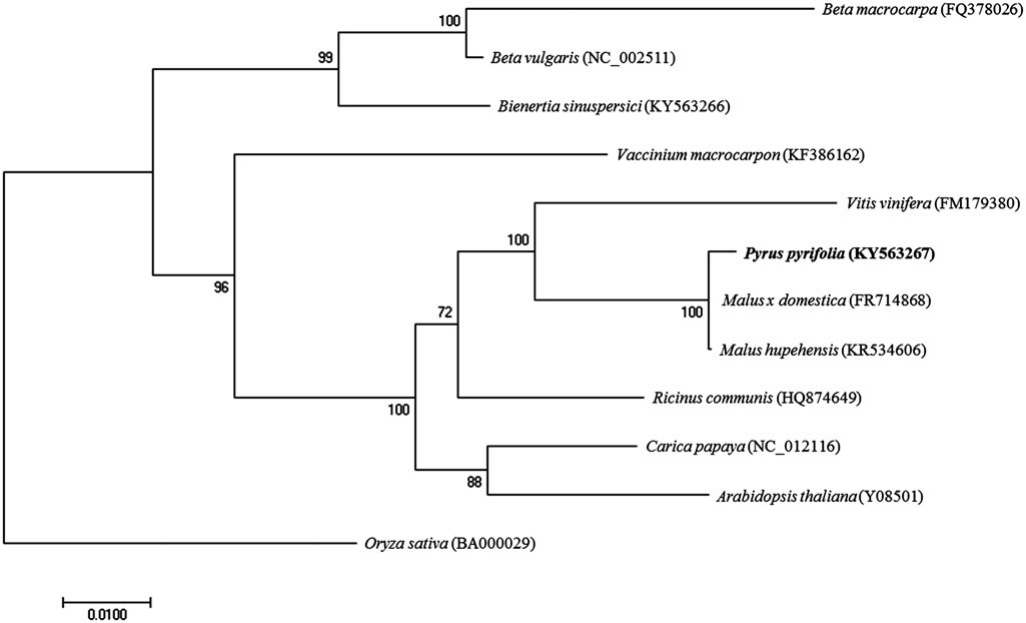
Maximum-likelihood tree of complete mitochondrial genome of *P. pyrifolia* and 11 other species. The numbers at the nodes are bootstrap percent probability values based on 1000 replications. Mitochondrial genome sequences were obtained from GenBank and their accession numbers are indicated next to species name.
